# Angiotensin II increases glomerular permeability by β-arrestin mediated nephrin endocytosis

**DOI:** 10.1038/srep39513

**Published:** 2016-12-22

**Authors:** Eva Königshausen, Ulf M. Zierhut, Martin Ruetze, Sebastian A. Potthoff, Johannes Stegbauer, Magdalena Woznowski, Ivo Quack, Lars C. Rump, Lorenz Sellin

**Affiliations:** 1 Nephrology, Medical School Duesseldorf, Heinrich Heine University, Moorenstr. 5, 40225 Duesseldorf, Germany

## Abstract

Glomerular permeability and subsequent albuminuria are early clinical markers for glomerular injury in hypertensive nephropathy. Albuminuria predicts mortality and cardiovascular morbidity. AT1 receptor blockers protect from albuminuria, cardiovascular morbidity and mortality. A blood pressure independent, molecular mechanism for angiotensin II (Ang II) dependent albuminuria has long been postulated. Albuminuria results from a defective glomerular filter. Nephrin is a major structural component of the glomerular slit diaphragm and its endocytosis is mediated by β-arrestin2. Ang II stimulation increases nephrin-β-arrestin2 binding, nephrin endocytosis and glomerular permeability in mice. This Ang II effect is mediated by AT1-receptors. AT1-receptor mutants identified G-protein signaling to be essential for this Ang II effect. Gαq knockdown and phospholipase C inhibition block Ang II mediated enhanced nephrin endocytosis. Nephrin Y1217 is the critical residue controlling nephrin binding to β-arrestin under Ang II stimulation. Nephrin Y1217 also mediates cytoskeletal anchoring to actin via nck2. Ang II stimulation decreases nephrin nck2 binding. We conclude that Ang II weakens the structural integrity of the slit diaphragm by increased nephrin endocytosis and decreased nephrin binding to nck2, which leads to increased glomerular permeability. This novel molecular mechanism of Ang II supports the use of AT1-receptor blockers to prevent albuminuria even in normotensives.

Albuminuria is a strong and independent predictor of cardiovascular mortality in the general population^1^,^2^. In patients with non-diabetic and diabetic kidney disease albuminuria is not only associated with cardiovascular mortality but also with progression to end-stage renal disease^3^. Inhibition of the renin angiotensin system (RAS) with angiotensin converting enzyme (ACE)-inhibitors or angiotensin-receptor blockers (ARB) effectively reduces and delays albuminuria^4^,^5^,^6^. Studies revealed that the anti-albuminuric effect of ACE-inhibitors and ARBs exceeded the benefit of blood pressure control alone^4^,^7^,^8^,^9^,^10^. ACE-inhibitors were shown to have the strongest anti-albuminuric effect under comparable blood pressure control when compared to calcium antagonists, diuretics and beta-blockers^10^. Therefore blood-pressure independent mechanisms for ACE-inhibitors and ARBs have been postulated to explain the renoprotective effects^5^,^11^.

Angiotensin II (Ang II) infusion in a non-blood pressure effective dose induces a significant transient increase in glomerular permeability^12^. The increased transient glomerular permeability under Ang II infusion without a significant rise of systemic blood pressure points to a blood pressure independent effect on the glomerular filtration barrier^13^,^14^.

The glomerular filtration barrier is composed of the three layers: the fenestrated endothelium, the glomerular basement membrane and the glomerular slit diaphragm formed in between the secondary podocyte foot processes. An essential component of the glomerular slit diaphragm is nephrin which is subjected to endocytosis by binding to β-arrestin2^15^. We and others have shown that endocytosis is crucial to podocyte integrity in development, health and disease^16^,^17^.

Previous studies have investigated the influence of Ang II on aspects of podocyte biology. Macconi *et al*. showed that Ang II stimulation in podocyte cell culture leads to an increased albumin permeability across podocyte monolayers and an Ang II mediated reorganization of the actin cytoskeleton^18^. Hsu *et al*. demonstrated the role of the activated Rac-1 on the remodeling of the F-actin cytoskeleton under Ang II stimulation of podocytes with stable AT1-receptor expression^19^. Greka and Mundel summarized their work on the influence of Ang II on the cytoarchitecture of the podocyte^20^, Yu *et al*. observed a significantly reduced nephrin phosphorylation *in vitro* after Ang II stimulation^21^. Ang II is also linked to proinflammatory states. Ayoub *et al*. described a functional interaction between the AT1-receptor and the chemokine receptor CCR2 which could be an interesting link between glomerular inflammation and proteinuria^22^.

In this study, we examined the influence of Ang II on nephrin endocytosis in order to identify the underlying signaling mechanisms that potentially contribute to blood pressure independent effects of ACE-inhibitors and ARBs on podocytes.

## Results

Here we show enhanced nephrin endocytosis by Ang II stimulation of podocytes. Nephrin endocytosis is responsible for the systemic blood pressure independent increase of glomerular permeability.

### Ang II increases glomerular permeability, enhances nephrin binding to β-arrestin2 and nephrin endocytosis

**Figure 1:**
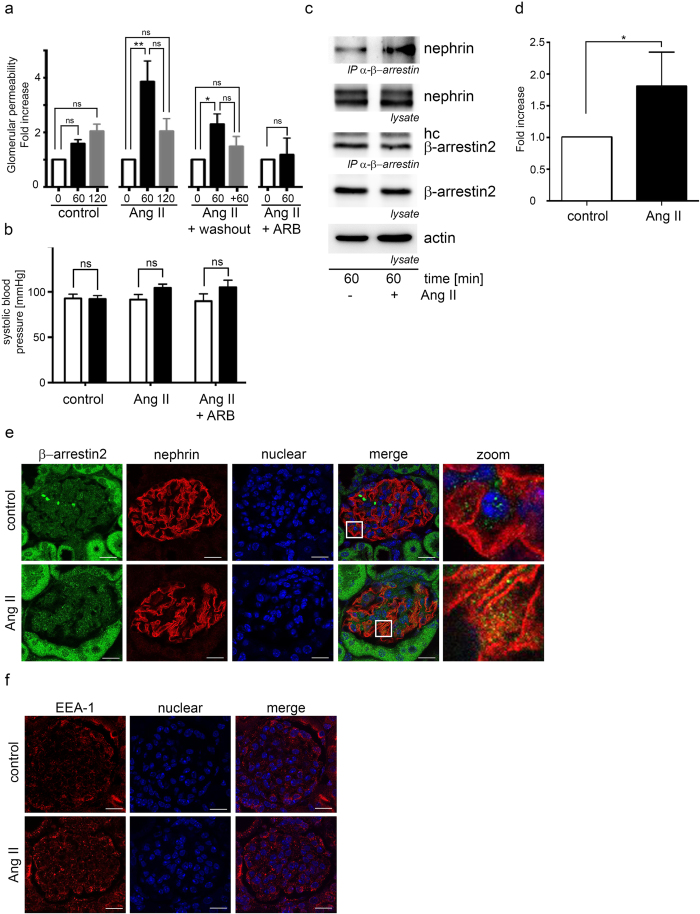
Ang II increases the glomerular permeability and enhances the interaction of β-arrestin to nephrin. (**a**) Ang II increases the glomerular permeability significantly in FVB mice (n = 5 per data point, p < 0.004 tested by Kruskal-Wallis test). The glomerular permeability was measured by FITC-Ficoll 70 appearance in the urine before (white columns, 0 min), 60 minutes after the begin of Ang II stimulation (black columns, 60 min) and after additional 60 minutes of Ang II stimulation or 60 minutes of Ang II discontinuation washout (grey columns, 120 min or +60 min). The measured FITC-Ficoll 70 concentrations in the urine were referenced to the urine creatinine concentration. Pretreatment with candesartan blocks the Ang II mediated increased glomerular permeability (control n = 9, 0 min vs 60 min p = 0.35, 60 min vs 120 min p = 0.87; Ang II n = 9, 0 min vs 60 min p = 0.0036, 60 min vs 120 min p = 0.18, 0 min vs 120 min p = 0.16; Ang II + washout n = 5, 0 min vs 60 min p = 0.038, 60 min vs 120 min p = 0.25, 0 min vs 120 min p = 0.58; Ang II + ARB n = 3, 0 min vs 60 min p = 0.99). (**b**) Systolic blood pressure was controlled by tail cuff measurement. No significant systolic blood pressure differences between the Ang II treated mice and the controls were found. (**c**) Mouse glomeruli were isolated from kidneys and stimulated with Ang II. Thereafter β-arrestin was immunoprecipitated from glomerular lysates and nephrin was shown to coimmunoprecipitate with β-arrestin. Ang II enhances the interaction of β-arrestin to nephrin significantly (n = 4, p = 0.03). Comparable protein amounts for β-arrestin, nephrin and actin were ensured by Western blotting. (hc – IgG heavy chain). (**d**) Densitometry of the by β-arrestin2 coimmunoprecitated nephrin. (**e**) Kidneys from mice treated with Ang II were stained for nephrin and β-arrestin2. The merged images from Ang II treated animals showed a pronounced colocalization of nephrin with β-arrestin2 after Ang II stimulation in comparison to the control. The scale bar represents 20 μm. (**f**) Kidneys from mice treated with Ang II were stained for EEA-1. Under Ang II stimulation an enhanced EEA-1 expression is noted. The scale bar represents 20 μm.

In mice the administration of Ang II for 60 minutes significantly increases the glomerular permeability in doses which do not influence blood pressure effectively (Fig. 1a + b). This effect is prevented by administration of an AT1 receptor blocker (Fig. 1a). After additional 60 minutes of Ang II washout the glomerular permeability decreases (Fig. 1a). The blood pressure was monitored by tail cuff measurements. No significant blood pressure changes were observed between the groups (Fig. 1b). Ang II mediated AT1 receptor signaling was controlled by increased phosphorylation of ERK (p42/p44) (Fig. 1Sa).

In isolated mouse glomeruli the stimulation with Ang II for 60 minutes enhances the β-arrestin binding to nephrin (Fig. 1c + d).

Ang II stimulation increases nephrin β-arrestin colocalization in mouse glomeruli (Fig. 1e). Due to previous data describing β-arrestin binding to nephrin as a mediator of nephrin endocytosis^15^ we looked for increased appearance of early endosomes in mouse glomeruli after stimulation with Ang II by EEA-1 staining. EEA-1 is a marker for early endosomes^23^. Ang II stimulation enhances endocytosis with increased appearance of EEA-1 positive vesicles (Fig. 1f).

**Figure 2:**
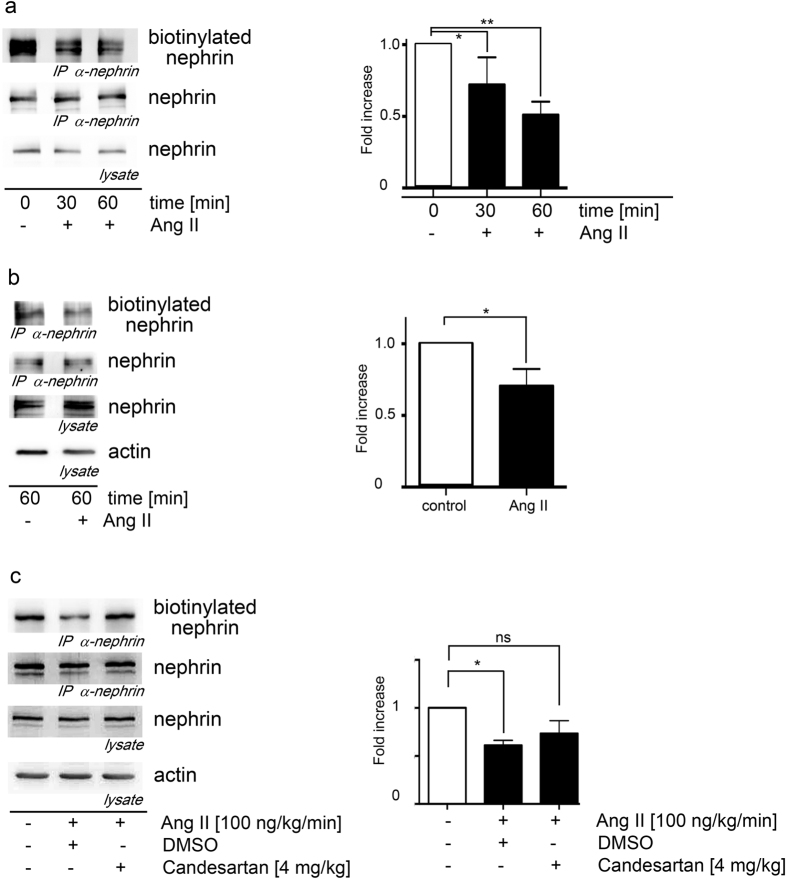
Ang II promotes nephrin endocytosis. (**a**) Ang II induces the endocytosis of nephrin in HEK293T cells significantly in a time dependent fashion. The biotinylated nephrin fraction decreases the longer the Ang II exposure lasts (Kruskal-Wallis test: *n = 5, p = 0.04; **n = 5, p = 0.009). The AT1-receptor plasmid was cotransfected in all conditions to the AT1-receptor deficient HEK293T cells. (**b**) Nephrin endocytosis in murine podocytes is significantly enhanced by Ang II (*n = 5, p = 0.03). Biotinylated nephrin is significantly decreased in mouse podocytes when they are stimulated with Ang II. (**c**) Nephrin endocytosis in mice without and with Ang II stimulation and additional candesartan treatment. Biotinylated nephrin is significantly decreased in Ang II treated animals compared to control mice and restored to nearly control levels by additional candesartan treatment. (*Kruskal-Wallis test: n = 5, p = 0.02).

In line with this, biotinylation assays show a significantly enhanced nephrin disappearance from the cell surface under Ang II stimulation in HEK293T cells and mouse podocytes (Fig. 2a + b). In mice the *in vivo* biotinylation of nephrin from glomerular extracts was shown to be reduced after 60 minutes of Ang II stimulation (Fig. 2c). This effect was blocked by the AT1 receptor blocker candesartan (Fig. 2c).

### AT1 receptor is essential for the Ang II mediated nephrin binding to β-arrestin2

**Figure 3:**
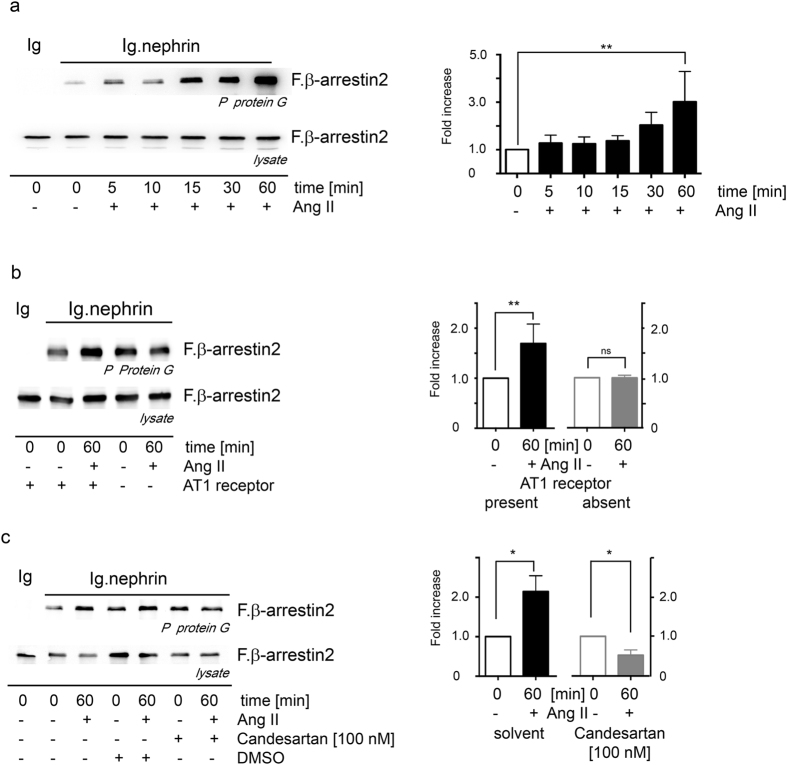
Ang II enhances the interaction of nephrin with β-arrestin2 through AT1 receptor activation. (**a**) HEK293T cells expressing nephrin, β-arrestin2 and the AT1-receptor were stimulated with Ang II. Ang II stimulation enhances the binding of β-arrestin2 to nephrin in a time dependent fashion. The maximum enhancement of 3-fold is seen after 60 min of Ang II stimulation (**Kruskal-Wallis test n = 12, p = 0.026). (**b**) HEK293T cells lacking the AT1 receptor do not show enhanced binding of nephrin to β-arrestin2 under stimulation with Ang II (**n = 9, p = 0.0019). The Ang II mediated enhanced binding of β-arrestin2 to nephrin is blocked by the AT1 receptor antagonist (**c**) Candesartan [100 nM] (*n  =  3, p = 0.03, ns = 0.314). Comparable amounts of nephrin fusion protein expression and its control were controlled by western blot (experiments were conducted in HEK293T cells). The expression of the transfected nephrin fusion proteins and its control is shown in the supplemental Fig. 1Sb–d. The Ang II mediated activation of ERK is shown in the supplemental Fig. 3Sa.

In HEK 293 T cells expressing the nephrin c-terminus, β-arrestin2 and AT1 receptor, an enhanced β-arrestin2 binding to nephrin is observed under Ang II stimulation. Ang II stimulation increases β-arrestin2 binding to nephrin c-terminus already after 5 minutes. This binding is time dependent and was the strongest at 60 min Ang II stimulation (Fig. 3a). The AT1 receptor is mandatory to enhance the β-arrestin2 binding to nephrin c-terminus under Ang II stimulation (Fig. 3b). In experiments with cells lacking the AT1 receptor the Ang II stimulation fails to induce the enhanced β-arrestin2 binding to the nephrin c-terminus. AT1 receptor antagonist candesartan blocked the enhanced β-arrestin2 binding to the nephrin c-terminus under Ang II stimulation (Fig. 3c).

The expression of the transfected nephrin fusion proteins and its control is shown in the supplemental Fig. 1Sb–d. The Ang II mediated activation of ERK is shown in the supplemental Fig. 3Sa.

### G-protein coupled signaling of the AT1 receptor and PLC enhance nephrin binding to β-arrestin2

**Figure 4:**
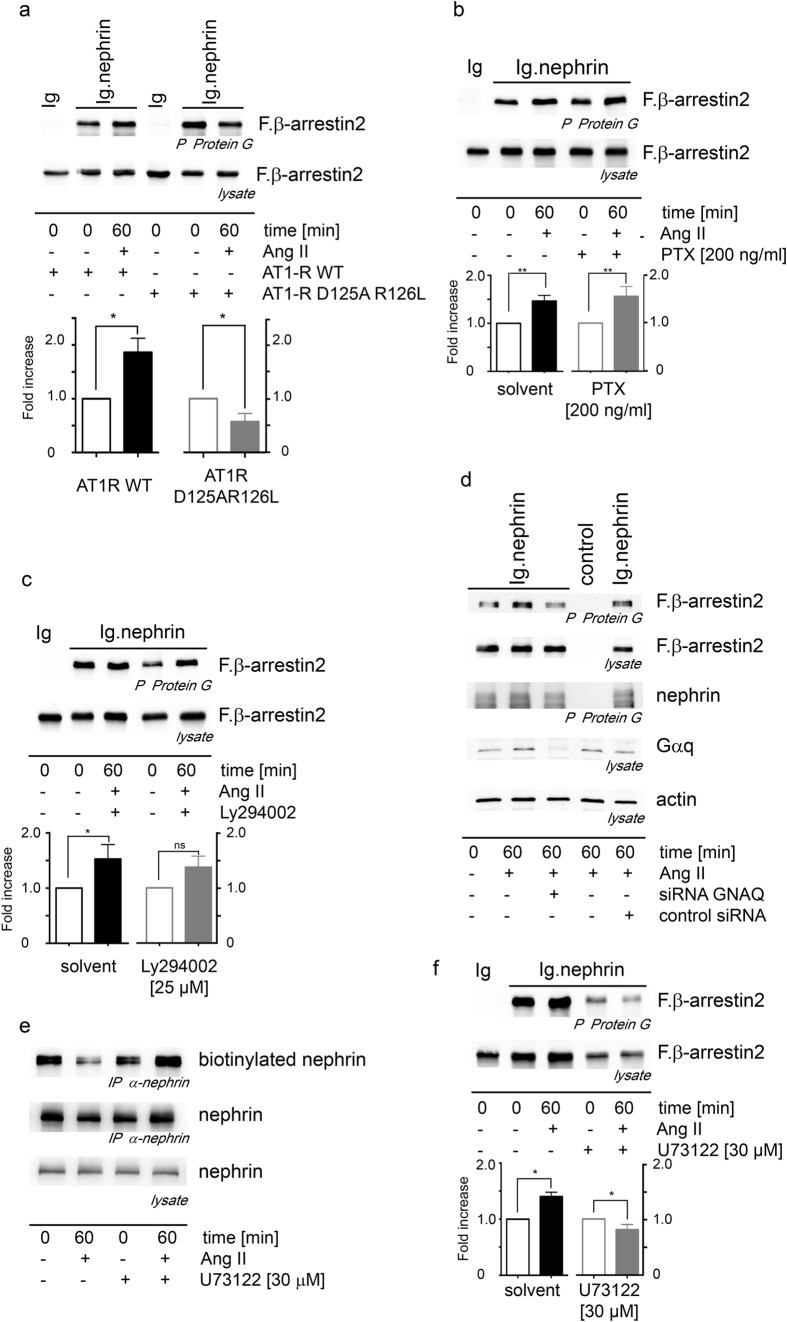
The Gα_i_ signaling is essential in conveying the Ang II mediated, enhanced β-arrestin2 binding to the nephrin-c-terminus and nephrin endocytosis. (**a**) The AT1 receptor D125AR126L mutant abolishes G-protein signaling and disables the Ang II induced enhanced β-arrestin2 binding to the nephrin-c-terminus (*n = 4, p = 0.014). (**b**) The inhibition of Gα_i_ by pertussis toxin (PTX) does not prevent the Ang II mediated enhanced β-arrestin2 binding to the nephrin-c-terminus (**n = 5, p = 0.0079). (**c**) The inhibition of the PI3kinase with Ly294002 does not influence the Ang II enhanced interaction of the nephrin-c-terminus-β-arrestin2 excluding the Gβγ pathway (*n = 4, p = 0.0143, p = 0.15 for Ly294002). (**d**) Gα_q_ mediates the AT1 receptor activation resulting in enhanced β-arrestin2 binding to the nephrin-c-terminus. The expression of GNAQ siRNA blocks the Ang II mediated enhancement of the β-arrestin2 binding to the nephrin-c-terminus. (**e**) The PLC inhibitor U73122 reduces the Ang II mediated nephrin endocytosis in HEK293T cells. (**f**) The PLC inhibitor U73122 abolishes the Ang II mediated enhanced β-arrestin2 binding to the nephrin-c-terminus in HEK293T (*n = 3, p = 0.0318). The use of the PLC inhibitor U73122 causes a reduced protein expression compared to conditions without U73122. All experiments were conducted in HEK293T cells. Comparable amounts of the nephrin-c-terminus fusion protein expression and its control were controlled by western blot. The expression of equivalent amounts of the AT1-receptors and its mutants was ensured by RT-PCR. The expression of the transfected nephrin fusion proteins and its control is shown in the supplemental Fig. 1Se–g. The expression of equivalent amounts of the AT1-receptors and its mutants was ensured by RT-PCR (supplemental Fig. 3Sb).

To get more insights into the relevant AT1 receptor mediated signaling in this context we used an AT1 receptor mutant (AT1-R D125A R126L) deficient for G-protein signaling. This mutant AT1 receptor failed to enhance the β-arrestin2 binding to nephrin under Ang II stimulation (Fig. 4a). The expression of the AT1 receptor mutants were ensured by RT-PCR (supplement Fig. 3Sb). We used inhibitors and siRNA to unravel the G-protein signaling responsible in conveying the signal from the activated AT1 receptor to the enhanced β-arrestin2 binding to nephrin. The use of PTX as an inhibitor of Gαi did not block the Ang II mediated enhanced β-arrestin2 binding to nephrin (Fig. 4b). The use of Ly294002 as a PI3 kinase inhibitor downstream of Gβγ failed to block the Ang II mediated enhanced β-arrestin2 binding to nephrin (Fig. 4c). As no suitable pharmacological inhibitors of Gαq are available we chose a siRNA knockdown approach. The efficient siRNA knockdown of Gαq inhibited the Ang II mediated enhancement of the β-arrestin2 binding to nephrin (Fig. 4d). PLC is downstream of Gαq in the signaling cascade. To further confirm the Gαq pathway of the activated AT1 receptor we used the PLC inhibitor U73122. The PLC inhibitor prevented the Ang II mediated enhancement of the β-arrestin2 binding to nephrin (Fig. 4f) and blocked the enhanced nephrin endocytosis under Ang II stimulation (Fig. 4e) confirming the Ang II mediated activations of the AT1-receptor and the signaling through Gαq and PLC. The use of the PLC inhibitor U73122 causes a reduced protein expression compared to conditions without U73122. The expression of the transfected nephrin fusion proteins and its control is shown in the supplemental Fig. 1Se–g. The expression of equivalent amounts of the AT1-receptors and its mutants was ensured by RT-PCR (supplemental Fig. 3Sb).

### Nephrin Y1217 is mandatory for the Ang II mediated enhanced binding of nephrin to β-arrestin2

**Figure 5:**
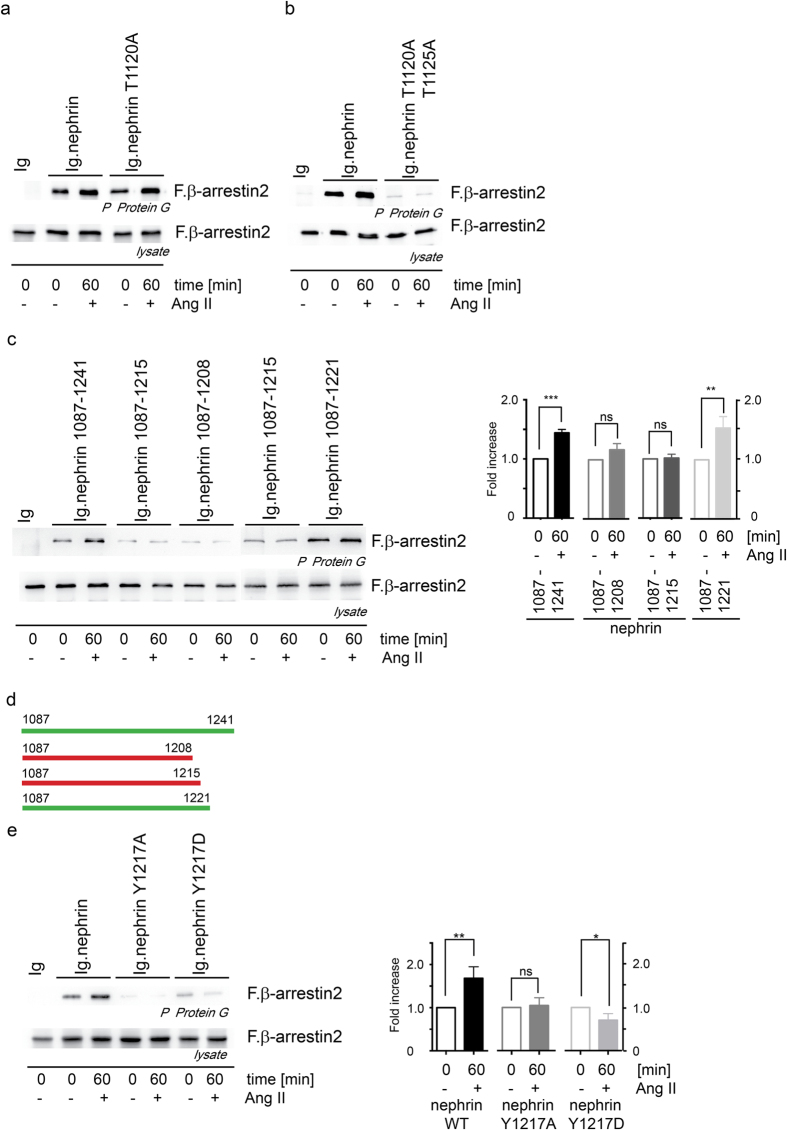
Nephrin Y1217 is critical for the Ang II mediated enhancement of the β-arrestin2 binding to nephrin. Several distinct nephrin residues have influence on the nephrin β-arrestin2 binding and the regulatory sensitivity to Ang II stimulation. The nephrin motif T-GERD-T at nephrin T1120/T1125 forms the β-arrestin2 binding site. (**a**) The nephrin T1120A mutant does not impair the binding of β -arrestin2 to nephrin and is still susceptible to Ang II. (**b**) The nephrin double mutant T1120A/T1125A impairs binding of β-arrestin2 completely. (**c**) The truncation mapping of the nephrin c-terminus unravels the nephrin aminoacid residues 1215–1221 to harbor the region that mediates the Ang II dependent enhanced β-arrestin2 binding to nephrin. The c-terminal nephrin truncations nephrin 1087–1221 show the Ang II mediated enhanced β-arrestin2 binding to nephrin whereas the nephrin truncations nephrin 1087–1208 and nephrin 1087–1215 lack the Ang II mediated enhanced β-arrestin2 binding to nephrin. P-values β-arrestin binding to nephrin for the comparison of Ang II stimulation versus without Ang II stimulation: Nephrin 1087–1241 n = 8, p = 0.0006; Nephrin 1087–1208 n = 4, p = 0.27; Nephrin 1087–1215 n = 6, p < 0.9; Nephrin 1087–1221 n = 5, p = 0,01 (**d**) Graphical summary of the nephrin c-terminal truncation mapping locating the transmission site of the Ang II mediated enhanced β-arrestin2 binding to nephrin. Green bars symbolize truncations which harbor the Ang II effect, red bars symbolize truncations that lack the Ang II effect. (**e**) The point mutations of nephrin Y1217 to an alanine or aspartatic acid allow the β-arrestin2 binding to nephrin but lack the Ang II mediated enhanced β-arrestin2 binding to nephrin (**n = 5, p = 0.004; *n = 5, p = 0.0476). All experiments were conducted in HEK293T cells. Comparable amounts of nephrin fusion protein expression and its control were controlled by western blot. The expression of the transfected nephrin fusion proteins and its control is shown in the supplemental Fig. 2Sa–d.

We further investigated the molecular mechanism within the nephrin c-terminus to elucidate how the Ang II treatment modifies nephrin’s capability to bind to β-arrestin2. Previous experiments showed that the β-arrestin2 binding site within nephrin is the T-GERD-T motif at nephrin 1120-1125^15^,^16^. Point mutations of nephrin T1120 and nephrin T1125 demonstrate the importance of both threonines for the nephrin–β-arrestin binding (Fig. 5a + b). The Ang II mediated enhanced nephrin-β-arrestin binding is not affected by the nephrin T1120A mutation (Fig. 5a). Only the double mutation of both threonines abolishes the nephrin-β-arrestin binding as the β-arrestin2-binding site is destroyed (Fig. 5b). The phosphorylation of nephrin T1120/T1125 by PKC is a mandatory prerequisite to mediate binding between nephrin and β-arrestin2. Truncation mapping of the nephrin c-terminus (Fig. 5c + d) helped to narrow the regulatory region for the Ang II mediated enhanced binding of β-arrestin2 to nephrin down to the six nephrin amino acids 1216–1221 (IYDQVA). This region contains also one of the three known nck binding sites. A point mutation of the relevant Y1217 to an alanine or aspartic acid allowed the binding of β-arrestin2 to nephrin but inhibited the Ang II mediated enhanced binding of β-arrestin2 to nephrin (Fig. 5e). Interestingly for nephrin Y1217 is a non-synonymous SNP (db SNP number: rs114879227) causing the tyrosine to aspartate mutation known.

The expression of the transfected nephrin fusion proteins and its control is shown in the supplemental Fig. 2Sa–d.

### Ang II attenuates nephrin binding to nck2

**Figure 6:**
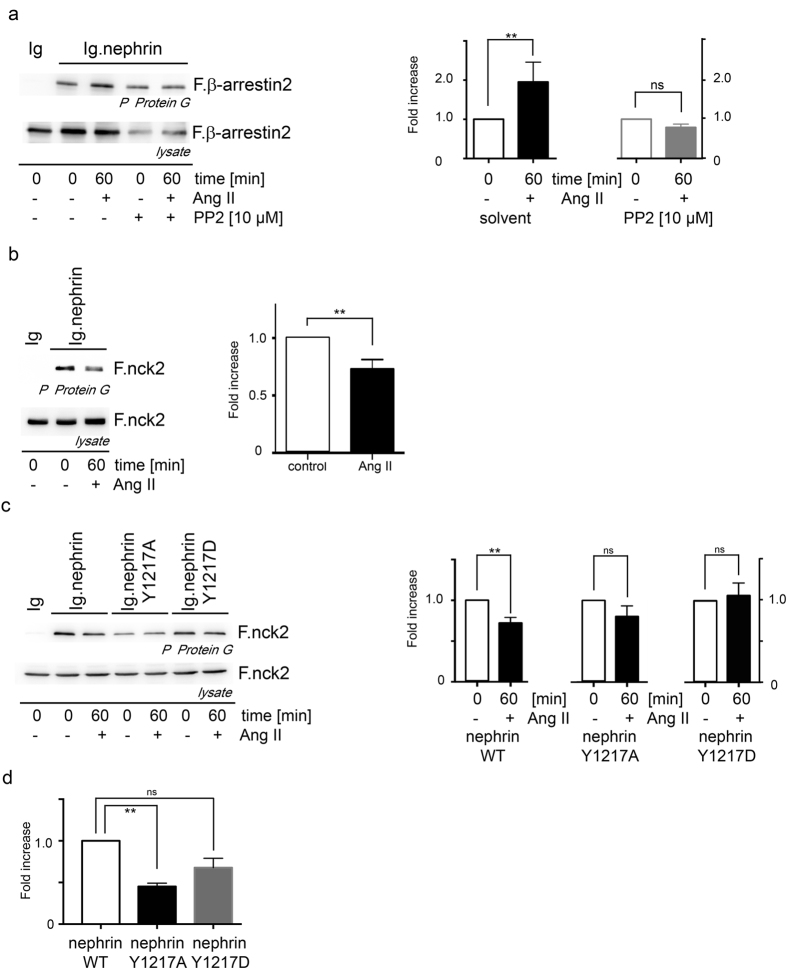
Ang II decreases nck2 binding to nephrin. (**a**) The tyrosine kinase inhibitor PP2 enhances the β-arrestin2 binding to nephrin under Ang II stimulation. PP2 alone without Ang II stimulation does not influence the β-arrestin2 binding to nephrin (**DMSO: n = 5, p = 0.004; PP2: n = 5, p = 0.0079). The use of the tyrosine kinase inhibitor PP2 causes a reduced protein expression compared to conditions without PP2. (**b**) Ang II stimulation attenuates nephrin nck2 binding (**n = 5, p = 0.0079). (**c**) Nephrin Y1217WT demonstrates under Ang II stimulation a significantly decreased binding to nck2 Y1217WT (n = 6, p < 0.008). The nephrin mutants Y1217A and Y1217D failed to demonstrate an effect to the nck2 binding to nephrin under Ang II stimulation. But the nephrin Y1217A mutant showed a significantly decreased binding to nck2 whereas the Y1217D mutant presented no different binding to nck2 than the nephrin. (**d**) Densitometry of nck2 binding to nephrin and its mutants without Ang II stimulation (n = 6, **p = 0.0023, ns p = 0.17). All experiments were performed in HEK293T cells. Comparable amounts of nephrin fusion protein expression and its control were controlled by western blot. The expression of the transfected nephrin fusion proteins and its control is shown in the supplemental Fig. 2Se–g.

Being aware of the tyrosine kinases’s role for β-arrestin2 binding to nephrin, the treatment with the tyrosine kinase inhibitor PP2 inhibits the Ang II mediated β-arrestin2 binding to nephrin (Fig. 6a). Knowing that Ang II promotes the nephrin endocytosis through an enhanced β-arrestin2 binding to nephrin we were curious what happens to known cytoskeletal anchoring structures of nephrin. Nck is a prominent and well described adaptor molecule of tyrosine phosphorylated nephrin that builds the link to the actin cytoskeleton and uses nephrin Y1217 as one interaction site^24^. The typical nck binding motif within nephrin is the aminoacid sequence LYDEV which appears three times within the human nephrin c-terminus. Only the mutation of all these three tyrosines within nephrin LYDEV motifs abolishes the nck binding to nephrin^24^,^25^. Interestingly the Ang II stimulation attenuates the nck2 binding to nephrin (Fig. 6b). By the use of nephrin Y1217 mutants the importance of this residue for nck2 binding is underlined. The nephrin WT shows a decreased interaction with nck2 under Ang II stimulation. The nephrin point mutants Y1217A and Y1217D lost the Ang II sensitivity (Fig. 6c). Independent of Ang II stimulation the nephrin Y1217A mutant shows significantly reduced binding to nck2, whereas the nephrin Y1217D mutant mimicking a constantly phosphorylated Y1217 residue binds at normal levels to nck2 without sensitivity to Ang II stimulation (Fig. 6c + d). This observation stresses the importance of the nephrin Y1217 residue for the nck2 interaction and underlines that the Ang II influence on the nephrin nck2 binding is conveyed by the phosphorylation change of the nephrin Y1217 (Fig. 6c). The expression of the transfected nephrin fusion proteins and its control is shown in the supplemental Fig. 2Se–g.

**Figure 7:**
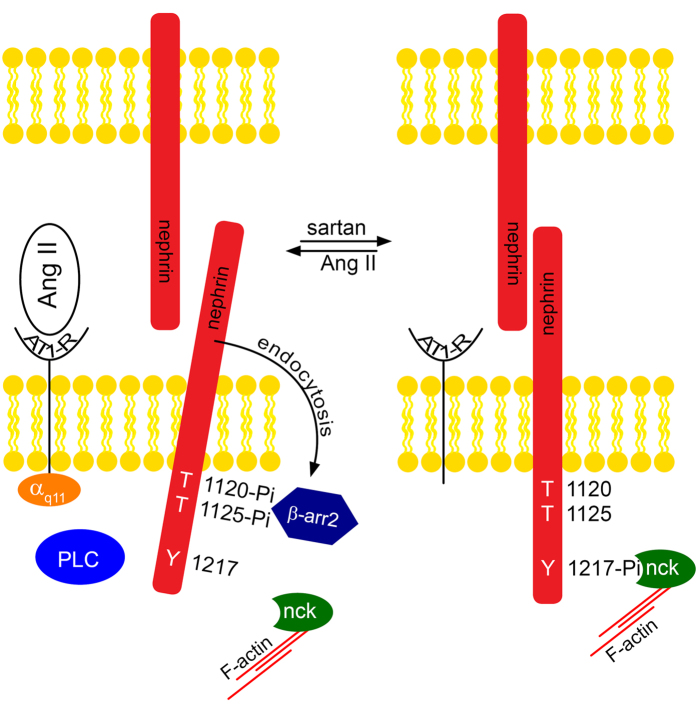
This scheme of function depicts how Ang II promotes glomerular permeability by nephrin endocytosis. Ang II activates the AT1-receptor (AT1-R). The activated AT1-receptor transfers the signal through G_αq/11_ and PLC. In turn nephrin gets phosphorylated at T1120/T1125 forming a β-arrestin2 binding site^16^. The binding of β-arrestin2 leads to increased nephrin endocytosis. This could be the mode of action how the glomerular permeability is increased beyond hemodynamic effects. In contrast, inhibition of the AT1-receptor by sartans promotes less β-arrestin2 nephrin interaction and less nephrin endocytosis leading to decreased glomerular permeability.

In summary we found an Ang II enhanced nephrin endocytosis mediated by β-arrestin2. At the same time the known interaction of nephrin to nck2 is alleviated under Ang II stimulation. One could speculate that Ang II functionally switches nephrin from its stabilizing nck interaction to the destabilizing interaction to β-arrestin2 (Fig. 7).

## Discussion

Previously Buter *et al*.^26^ showed that initiation of losartan treatment in diabetic patients reduces urine albumin excretion within 3 days without a significant blood pressure reduction. After discontinuation of losartan the anti-albuminuric effect disappeared. Furthermore, Axelsson *et al*. demonstrated a significant rise of glomerular permeability within minutes after Ang II stimulation. This Ang II infusion showed no significant change of the systemic blood pressure^12^. The Ang II mediated rapid increase in glomerular permeability is reversible within several minutes after Ang II stimulation^12^. We also observed a rapid response to Ang II stimulation leading to enhanced β-arrestin2 binding to nephrin within minutes and subsequent nephrin endocytosis. In our animal model we also observed a decrease of the Ang II mediated glomerular permeability after 60 minutes of Ang II washout. However, the permeability decrease was not significantly different versus the control nor the Ang II treatment. It is debatable whether 60 minutes of washout are sufficient to fully reverse the Ang II mediated increase of the glomerular permeability. This is a novel molecular mechanism to describe the rapid increase of glomerular permeability by Ang II stimulation. It is without question that significant decrease in systemic blood pressure and influence on the intraglomerular hemodynamics will decrease albuminuria^27^. Independent of the systemic blood pressure ARB treatment significantly reduced albuminuria whereas a calcium antagonist was potent to control the blood pressure but failed to reduce the albuminuria^28^. Despite possible different effects on intraglomerular hemodynamics one might attribute something unique to Ang II inhibition conveying an additional anti-albuminuric effect. The Ang II enhanced binding to nephrin and subsequent nephrin endocytosis provides a novel molecular mechanism to explain the anti-albuminuric moiety of Ang II inhibition beyond hemodynamic effects. This novel mechanism supports the clinical observation that the pharmacologic inhibition of Ang II reduces albuminuria in normotensive albuminuric patients. From previous work we know that nephrin tyrosine phosphorylation regulates the nephrin binding to β-arrestin2^15^,^17^ as well as to nck^24^. For the nephrin β-arrestin2 interaction the nephrin tyrosine 1193 dephosphorylation was critical. This previous work also showed the β-arrestin2 interaction to nephrin being attenuated in presence of enhanced tyrosine kinase activity (Yes)^15^. The additional use of the tyrosine kinase inhibitor PP2 neutralized the effect of the tyrosine kinase (Yes) on the nephrin β-arrestin2 interaction^15^. Here the use of PP2 inhibits the tyrosine phosphorylation of nephrin and thereby enhances the binding of β-arrestin2 to nephrin and diminishes the binding of nck to nephrin. The PP2 effect of diminished nephrin binding to β-arrestin2 can be in part functionally antagonized by Ang II stimulation.

For the nephrin nck binding the nephrin tyrosine phosphorylation of Y1176, 1193, 1217 was essential^24^. Here our results underline the stabilizing effect of nephrin tyrosine 1217 phosphorylation favoring the binding to nck and at the same time reducing the binding to β-arrestin2.

In the human 1000 genome population the nephrin tyrosine 1217 is a non-synonymous SNP (rs 114879227, Y1217D) with a heterozygosity frequency of 0.009^29^. It would be interesting to know if the carriers of this SNP are protected against Ang II mediated albuminuria. Interestingly in clinical situations the use of the tyrosine kinase inihibitors ramucirunab, sorafinib and sunitinib could cause reversible albuminuria as a side effect^30^,^31^,^32^,^33^ underlining the clinical relevance of tyrosine dephosphorylation for albuminuria.

One might ask whether the nephrin endocytosis causes the increased glomerular permeability or the nephrin endocytosis is the consequence of increased glomerular permeability. Further work will be needed to answer this question.

It needs to be mentioned that cell surface disappearance of biotinylated nephrin could also be caused by extracellular nephrin shedding as it has been observed in a podocyte cell culture based study^34^. If nephrin shedding does occur a truncated nephrin can be detected. In our study we could not detect truncated nephrin. Therefore we expect the nephrin endocytosis to be the cause of the nephrin cell surface disappearance.

In summary, we could demonstrate a molecular mechanism for Ang II increasing glomerular permeability and paving the track for albuminuria beyond hemodynamic effects. The relevant AT1-receptor signaling and its downstream messengers were unraveled. Within nephrin the critical residue controlling between endocytosis and the stabilizing linkage to the actin cytoskeleton was identified. This opened the perspective for a regulatory model: AT1 receptor activation by Ang II promotes glomerular permeability and mediates nephrin endocytosis. On the other hand AT1 receptor blockade favors nephrin binding to nck linking nephrin to the actin cytoskeleton.

## Materials and Methods

All of the reagents were purchased from Sigma Aldrich (Munich, Germany) unless stated otherwise. Candesartan (CV 11974) was a kind gift of Astra Zeneca (Wedel, Germany). PP2 (tyrosine kinase inhibitor), U73122 (PLC inhibitor) and Ly294002 (PI3 kinase inhibitor) were purchased from Calbiochem (Merck, Darmstadt, Germany). PTX was obtained from List Biological Laboratories (Campbell, CA, USA) and N-hydroxysulfosuccinimydyl-SS-biotin was purchased from Pierce (Bonn, Germany); siRNA and transfection reagents (SMARTpool: ON-TARGETplus GNAQ siRNA L-008562-00-0005 and ON-TARGETplus Non-targeting Pool D-001810-10-05 as control, Dharmacon, Cologne, Germany) were obtained from Thermo Scientific and Dynabeads from Invitrogen (Darmstadt, Germany). Gamma bind plus sepharose was purchased from GE healthcare (Muenchen, Germany).

### Plasmids

Human nephrin cDNA was a gift from Dr. Gerd Walz (University of Freiburg, Germany). Membrane-bound fusion proteins of the C-terminal cytoplasmic domains of nephrin (AA 1087–1241, 1087–1215, 1158–1215, 1158–1241, 1087–1208, 1087–1221, 1087–1228) were generated by PCR and using a pCDM8 cassette that contained the leader sequence of CD5 fused to the CH2 and CH3 domains of human IgG1, followed by the transmembrane region of CD7 (Ig.nephrin)^35^. In brief, PCR was performed with the primers mentioned. The PCR product was purified by phenol extraction and further treated with restriction enzyme *Dpn I* and thereafter transformed into competent bacterial cells^36^. The Y1217A nephrin mutant (forward primer 5′cgcgggacgcgtcgcgggacgcgtcagcggagactcagg3′, reverse primer 5′gcgggggcggccgccgcgggggcggccgccttacaccagatgtcccctcagctcgaagggcagagaatcgggttccagagtgtccaagtctccggccacctggtccgcgattcctcttggatcc-3′) and the Y1217D nephrin mutant (5′gcgggggccgccttacaccagatgtcccctcagctcgaagggcagagaatcgggttccagagtgtcc-aagtctccggccacctggtcatcgattcctcttggatc-3′) were generated by PCR and cloned *MluI*/*NotI* into pCDM8 vector (see C-terminal cytoplasmic domain above). Clones were verified by sequencing.

FLAG-tagged β-arrestin2 (F.β-arrestin2) was a generous gift from Dr. Robert Lefkowitz^3^ while GFP-tagged AT1-receptor was provided by Dr. László Hunyady (Semmelweis University, Budapest, Hungary). Dr. Ying-Hong Feng (Uniformed Services University of the Health Sciences, Bethesda, MD) provided the AT1-receptor mutant (D125AR126L) which lacks G-protein coupled receptor signaling^37^. PCR from FLAG-tagged Nck2, which was a generous gift from Dr. Nina Jones (University of Guelph, Guelph, Canada), was performed and cloned *MluI*/*NotI* into the pCDM8 vector^24^.

### Cell Culture

Immortalized murine podocytes were generously provided by Dr. Peter Mundel (Massachusetts General Hospital, Boston, MA). The podocytes were grown on type I collagen under permissive temperature (33 °C) in the presence of 10 units/ml IFN-γ. To induce differentiation, the cells were maintained at 37 °C without IFN-γ for 10–14 days^38^.

AT1-receptor expressing murine podocytes were generated by retroviral transduction. The AT1-receptor was cloned into the pLEGFP-C1 vector *HindIII*/*NotI* via PCR with the following primers: 5′cgcgggaagcttatggcccttaactcttc and 5′ gcgggggcggccgcctcactccacctcaaaac. Briefly, infectious viral supernatants - containing the sequence verified AT1-receptor cDNA or the GFP control - were produced by transient transfection of HEK293T cells. Immortalized murine podocytes were infected with viral supernatants containing either the AT1-receptor or a GFP control for 48 h. Selection of transduced cells was performed by G418 (200 μg/ml). The expression of the AT1-receptor was confirmed by *q*-PCR and activation of the AT1-receptor signaling pathway p42/44 by Angiotensin II (Ang II) was confirmed by Western blot.

HEK293T cells were grown in DMEM/F-12 medium supplemented with FCS.

### Antibodies

The following antibodies were used: mouse anti-M2 and mouse anti-actin (Sigma Aldrich, Taufkirchen, Germany), mouse anti-V5 (Invitrogen, Karlsruhe, Germany), sheep anti-human IgG (GE Healthcare, Freiburg, Germany), rabbit anti-GNAQ (Santa Cruz Biotechnology, Santa Cruz, CA), guinea pig anti-Nephrin (Progen Biotechnik, Heidelberg, Germany), rabbit β-arrestin1/2 (Cell Signaling Technology, Frankfurt, Germany), rabbit anti-p42/44 und rabbit anti-phospho p42/44 (Cell Signaling, Leiden, Netherlands), streptavidin-HRP (Thermo Scientific, Darmstadt, Germany), protein A and G sepharose (GE, Freiburg, Germany). As secondary antibodies for Western blotting anti-mouse-HRP (Dako, Hamburg, Germany), anti-guinea pig-HRP (Millipore, Darmstadt, Germany) and anti-rabbit-HRP (GE, Freiburg, Germany) were used. Antibodies used for immunofluorescence are indicated below.

### Coimmunoprecipitation

Coimmunoprecipitation experiments were carried out as described before^35^. Briefly, HEK293T cells were transiently transfected by using the calcium phosphate method. After incubation for 24 h, the cells were stimulated with Ang II (1 μM) for the time indicated and harvested thereafter. For inhibitor experiments, cells were pretreated with the inhibitor or its control (Candesartan 100 nM, PP2 10 μM, PTX 200 ng/ml, Ly294002 25 μM, U73122 30 μM) for 60 min and stimulated with Ang II 1 μM thereafter. After cell lysis in 1% Triton X-100 lysis buffer, centrifugation (20000 × g, 15 min, 4 °C) was performed. Cell lysates containing equal amounts of total protein were incubated 1 h at 4 °C with protein G sepharose and extensively washed afterwards with lysis buffer. The precipitated proteins were resolved by 10% SDS-PAGE and visualized by Western blotting.

### siRNA knockdown

Cotransfection of HEK293T cells with specific siRNA and cDNA plasmids was performed according the manufacturer’s protocol. In brief, the cells were seeded into 6 cm tissue culture dishes in low glucose (5.5 mM) cell culture media. The next day cells were transfected with 2 μg DNA and 0.4 nmol of (control or target) siRNA in 4 ml transfection medium. After 24 h, the cells were equally divided into several dishes. HEK293T cells were treated with Ang II (1 μM, 60 min) or H_2_O 72 h after transfection before coimmunoprecipitation was performed. If an inhibitor was used, cells were pretreated with the inhibitor 60 min before stimulation with Ang II (1 μM).

### Biotinylation Assay

HEK293T cells were transfected with human nephrin cDNA. Before the cells were harvested in ice-cold PBS buffer containing 0.1 mM CaCl_2_ and 1 mM MgCl_2_ (PBSCM – phosphate buffered saline with CaCl_2_ and MgCl_2_), pH 8.0, cells were incubated with Ang II (1 μM, 60 min) or H_2_O. For inhibitor experiments, cells were pretreated with the inhibitor 60 min before stimulation with Ang II. Plasma membrane proteins were labeled with N-hydroxysulfosuccinimydyl-SS-biotin (0.5 mg/ml; Pierce) for 30 min at 4 °C. Unbound biotin was quenched twice with ice-cold PBSCM containing 100 mM glycine. Cells were lysed in 1% Triton X100 lysis buffer and nephrin was precipitated with GP-N2 antibody from Progen Biotechnik, Heidelberg, Germany. After extensive washing, cell lysates and bound proteins were resolved in 10% SDS and visualized via Western blotting.

For endogenous immunoprecipitation differentiated AT-1 receptor murine podocytes (8 × 19 × 10^5^ cells/data point) were incubated with Vitamin D3 (100 nM) as described elsewhere^39^. Before the process of biotin labelling (as indicated above) and cell lysis in Triton 1% lysis buffer podocytes were stimulated with Ang II or H_2_O (1 μM, 60 min), centrifuged (20.000 × g, 30 min, 4 °C) and equal amounts of protein were incubated with an anti-nephrin antibody over night at 4 °C. Afterwards cell lysates were incubated with protein A sepharose for 3 h at 4 °C followed by extensive washing and protein denaturation by SDS.

For immunfluorescence of mouse kidneys, mice were treated with a continuous infusion of Nacl 0,9% or Ang II (100ng/kg/min) via a central venous catheter. After 60 min, mice were sacrificed and their kidneys perfused with ice-cold PBS. Kidneys were fixed in paraformaldeyd. Slides were deparaffinized, pretreated with citrate buffer (pH 6.1) for 15 min at 98 °C and blocked with biotin block and protein block (Dako, Hamburg, Germany). Anti-β-arrestin 2 (Cell Signaling technology, Frankfurt, Germany 1:50) and anti-nephrin (Progen Biotechnik, Heidelberg, Germany 1:100) antibodies were incubated at 4 °C over night. For β-arrestin2 staining, enhancement with a biotinylated anti-rabbit antibody (Dianova, Hamburg, Germany 1:1000) was followed by incubation with streptavidin-Cy2 (Dianova, Hamburg, Germany 1:500). Nephrin was visualized via anti-guinea pig Alexa Flour 647 (Dianova, Hamburg, Germany 1:1000). Anti-EEA-1 (Santa Cruz Biotechnology, Santa Cruz, CA, USA 1:100) was stained with a biotinylated secondary rabbit antibody (Dianova, Hamburg, Germany 1:1000) and visualized with Streptavidin Cy3 (Dianova, Hamburg, Germany 1:1000). Mounting medium with Dapi was used (Dianova, Hamburg, Germany).

Samples were analyzed on a Leica TCS SP2 confocal laser-scanning microscope using a 63x HCX PL Apo water immersion objective (Leica, Wetzlar, Germany).

### Animal care

Mice were obtained from the local animal care facility. The investigations were conducted according the guidelines outlined in the Guide for Care and Use of Laboratory Animals (US National Institutes of Health Publication No. 85–23, revised 1996). All animal experiments were performed in accordance and compliance with the relevant institutional approvals (state government LANUV reference number: 84-02.04.2012.A397).

### Glomerular permeability measurement in mice

Female FVB mice at the age of 6–8 weeks were anesthetized. A central venous catheter was placed while urine was collected continuously. A bolus of FITC-Ficoll 70 (40 μg) was applied intravenously followed by a continuous infusion of 8 μl/min of FITC-Ficoll 70 (20 μg/ml in 0.9% NaCl). DMSO or CV11974 (4 mg/kg) were administered i.p. at this point. After 60 minutes of equilibration, continuous infusion of Ang II (100 ng/kg/min) (Ang II or Ang II + washout) or NaCl 0.9% (control) was started additionally. Urine collection was performed at 60 and 120 minutes thereafter. For the AngII washout experiment, continuous infusion of Ang II (Ang II) or Nacl 0,9% (control and Ang II + washout) was continued for another 60 minutes. Blood pressure measurement was performed every 60 minutes via the tail cuff method (Softron BP-98A, Japan). Urine was subjected to fluorescence measurement at 490 nm and creatinine was analyzed via an enzymatic assay following the manufactures instructions (Sigma Aldrich, Taufkirchen, Germany). Data were displayed as FITC/creatinine ratios at the end of the equilibration time (0 min) vs. the end of the experiment (60 min). Statistics were performed using 2-way ANOVA.

For *in vivo* endocytosis, mice were sacrificed and perfused with PBSCM, followed by biotin and glycine. Glomeruli were isolated and lysed as described below. Cell lysates were adjusted using the BCA method. Nephrin was precipitated with an anti-nephrin (Progen) antibody (5 μl) at 4 °C over night followed by 30 μl of protein A sepharose. After extensive washing of the precipitates, Western blot was performed under reducing conditions.

### Glomeruli isolation

Balb/c mice (male, 6–8 weeks of age) were sacrificed and their kidneys perfused with ice-cold PBS via the abdominal aorta. Thereafter the kidneys were perfused with Dynabeads (diameter: 4.5 μM; Invitrogen) at a concentration of 1.2 × 10^7 ^beads/ml in PBS. The kidneys were removed, minced, and digested with collagenase A (Roche Applied Science, Mannheim, Germany) for 30 min at 37 °C. The digested kidney tissue was sieved through a 100 μM cell mesh with intermittent PBS flushing. After centrifugation (620 × g, 5 min, 4 °C) the cell pellet was dissolved in 2 ml of PBS and transferred into a 2 ml tube. By using a magnet catcher and subsequent washing procedure, the Dynabeads containing glomeruli were washed until an appropriate purity of 95% was achieved. Isolated glomeruli were stimulated with Ang II (1 μM) for 60 min and lysed on ice in CHAPS buffer (20 mM CHAPS, 20 mM Tris pH 7,5, 50 mM NaCl, 50 mM NaF, 15 mM Na_4_P_2_O_7_, 0,1 mM EDTA pH 8,0, 2 mM sodiumorthovanadate, 2 mM ATP) by using a TissueRuptor (Quiagen, Hilden, Germany). Insoluble cellular material was removed by centrifugation (20.000 × g, 4 °C, 30 min). The resulting cell lysates were adjusted to ensure equal total protein content using the BCA method. After addition of an anti-β-arrestin antibody (1:50) the cell lysates were incubated over night at 4 °C followed by the incubation with 30 μl of Gamma bind plus sepharose for 3 h. The immunoprecipitates were washed extensively with lysis buffer, and the precipitated proteins were resolved by 10% SDS-PAGE under non-reducing conditions and visualized by Western blotting.

## Statistics

If not otherwise stated Mann-U-Whitney test was used (Graph Pad, Prism, Version 6a).

## Additional Information

**How to cite this article**: Königshausen, E. *et al*. Angiotensin II increases glomerular permeability by β-arrestin mediated nephrin endocytosis. *Sci. Rep.* **6**, 39513; doi: 10.1038/srep39513 (2016).

**Publisher's note:** Springer Nature remains neutral with regard to jurisdictional claims in published maps and institutional affiliations.

## Supplementary Material

Supplementary Information
