# Supplementary material for: Angiotensin II increases glomerular permeability by β-arrestin mediated nephrin endocytosis
Source: Sci Rep. 2016 Dec 22;6:39513. doi: 10.1038/srep39513 (PMC5177899; doi:10.1038/srep39513)
Supplement: Supplementary Information [file srep39513-s1.doc]

**Supplementary Material**

***Title:*** Angiotensin II increases glomerular permeability by β-arrestin mediated nephrin endocytosis

Eva Königshausen, MD^1^, Ulf M. Zierhut, MD^1^, Martin Ruetze, MD^1^, Sebastian A. Potthoff, MD^1^, Johannes Stegbauer, MD^1^, Magdalena Woznowski, MD^1^, Ivo Quack, MD^1^, Lars C. Rump, MD^1^, Lorenz Sellin, MD^1, *^

**Supplemental figure Legends**

**Figure 1S**

(a) Ang II mediated AT1 receptor signaling was involved in increased phosphorylation of ERK (p42/p44). Cell lysates from isolated glomeruli were separated by SDS-PAGE and subjected to western blotting. An antibody for total and phosphospecific p42/p44 detected respectively. Ang II mediates enhanced phosphorylation of p42/p44.

(b – g) Expression controls for the heterologous nephrin fusion protein in experiments with Ig.nephrin expression in cultured cells. Ig.nephrin consists out of an extracellular Ig-domain fused in frame to the fulllength cytosolic nephrin terminus. An anti-human IgG antibody recognizes the extracellular domain of Ig.nephrin as it contains the CH2 and CH3 domain of the human IgG1. This IgG domain is glycosylated and appears therefore as a broadened band/smear. Especially the negative control (Ig alone) is heavily glycosylated. P protein G stands for pulldown with protein G which binds to the IgG domain of Ig.nephrin. The expression of the Ig-fusion protein without the nephrin c-terminus (Ig alone) serves as a negative control.

**Figure 2S**

(a - c) Expression controls for the heterologous nephrin fusion protein in experiments with Ig.nephrin expression in cultured cells. Ig.nephrin truncations and mutants consist out of an extracellular Ig-domain fused in frame to the fulllength cytosolic nephrin c-terminus truncation as indicated by the amino acid numbers in the figures. (d) Expression controls for the heterologous nephrin fusion protein in experiments with Ig.nephrin WT and its Y1217 point mutant to alanine (Y1217A) and to aspartatic acid (Y1217D) in cultured cells. (e – g) Expression controls for the heterologous nephrin fusion protein in experiments with Ig.nephrin expression in cultured cells. Ig.nephrin consists out of an extracellular Ig-domain fused in frame to the fulllength cytosolic nephrin terminus. An anti-human IgG antibody recognizes the extracellular domain of Ig.nephrin as it contains the CH2 and CH3 domain of the human IgG1. This IgG domain is glycosylated and appears therefore as a broadened band/smear. Especially the negative control (Ig alone) is heavily glycosylated. P protein G stands for pulldown with protein G which binds to the IgG domain of Ig.nephrin. The expression of the Ig-fusion protein without the nephrin c-terminus (Ig alone) serves as a negative control.

**Figure 3S**

(a) Activation of ERK1/2 through AT1 receptor activation by Ang II in AT1-receptor overexpressing HEK293T cells 60 and 120 minutes after Ang II stimulation. (b) Expression control of the WT and mutant AT1-receptor. The mutant AT1-receptor is deficient for G-protein coupled signaling and not gfp-tagged as the WT control. RT-PCR for the AT1-receptor showed comparable transcript levels for the WT and mutant AT1-receptor normalized to GAPDH.

**Supplement Figure 1**

**
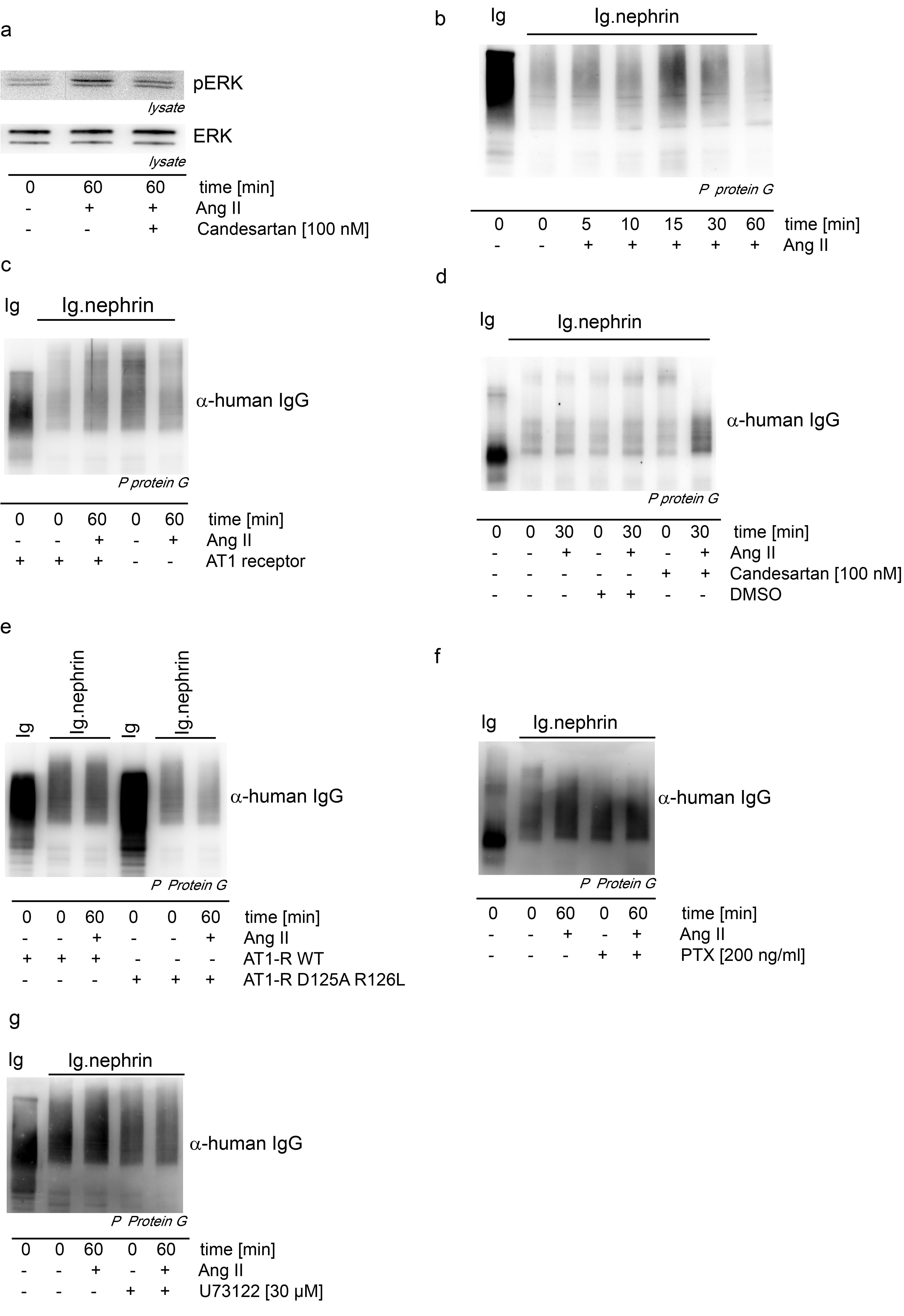
**

**Supplement Figure 2**

**
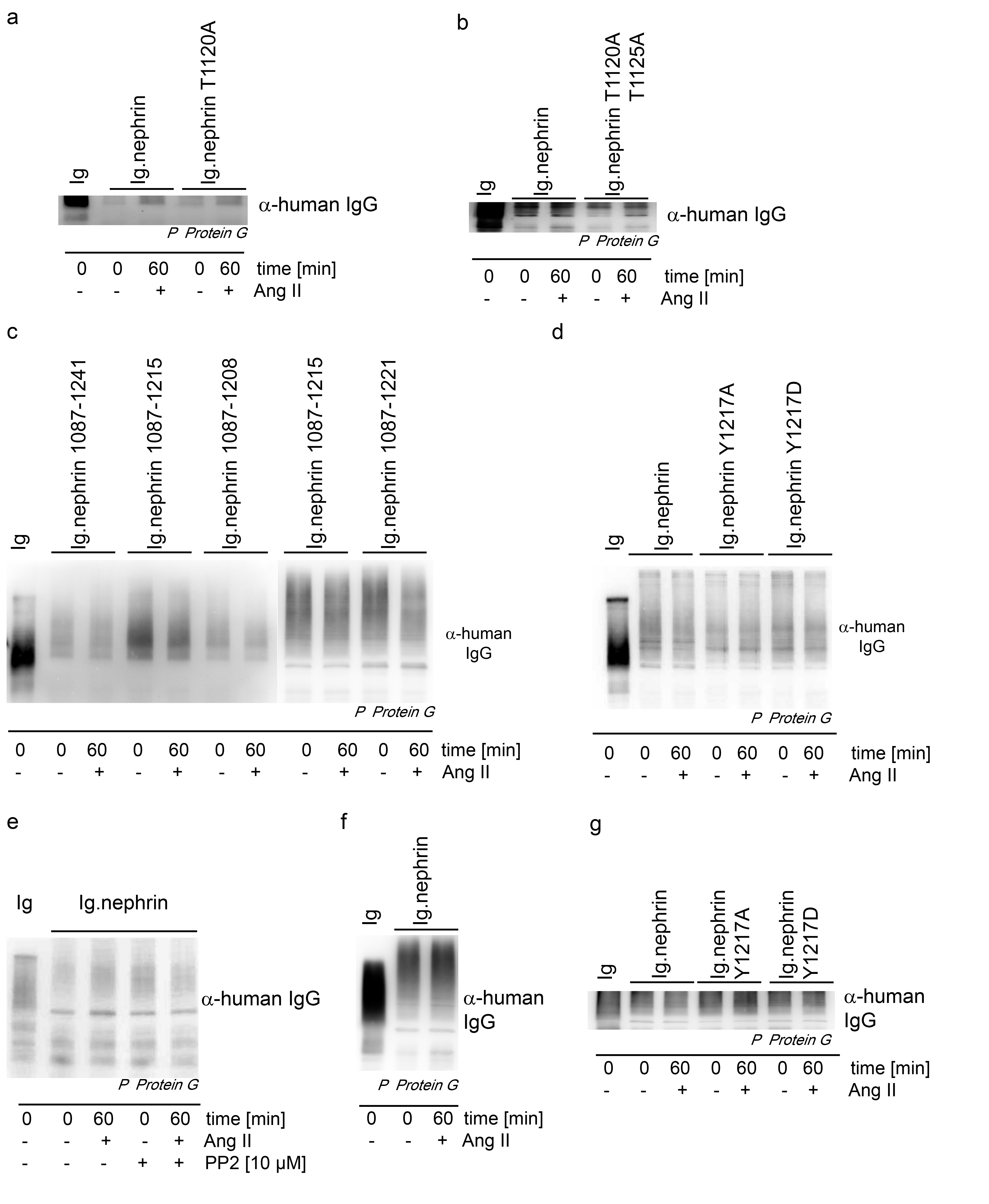
**

**Supplement Figure 3**

**
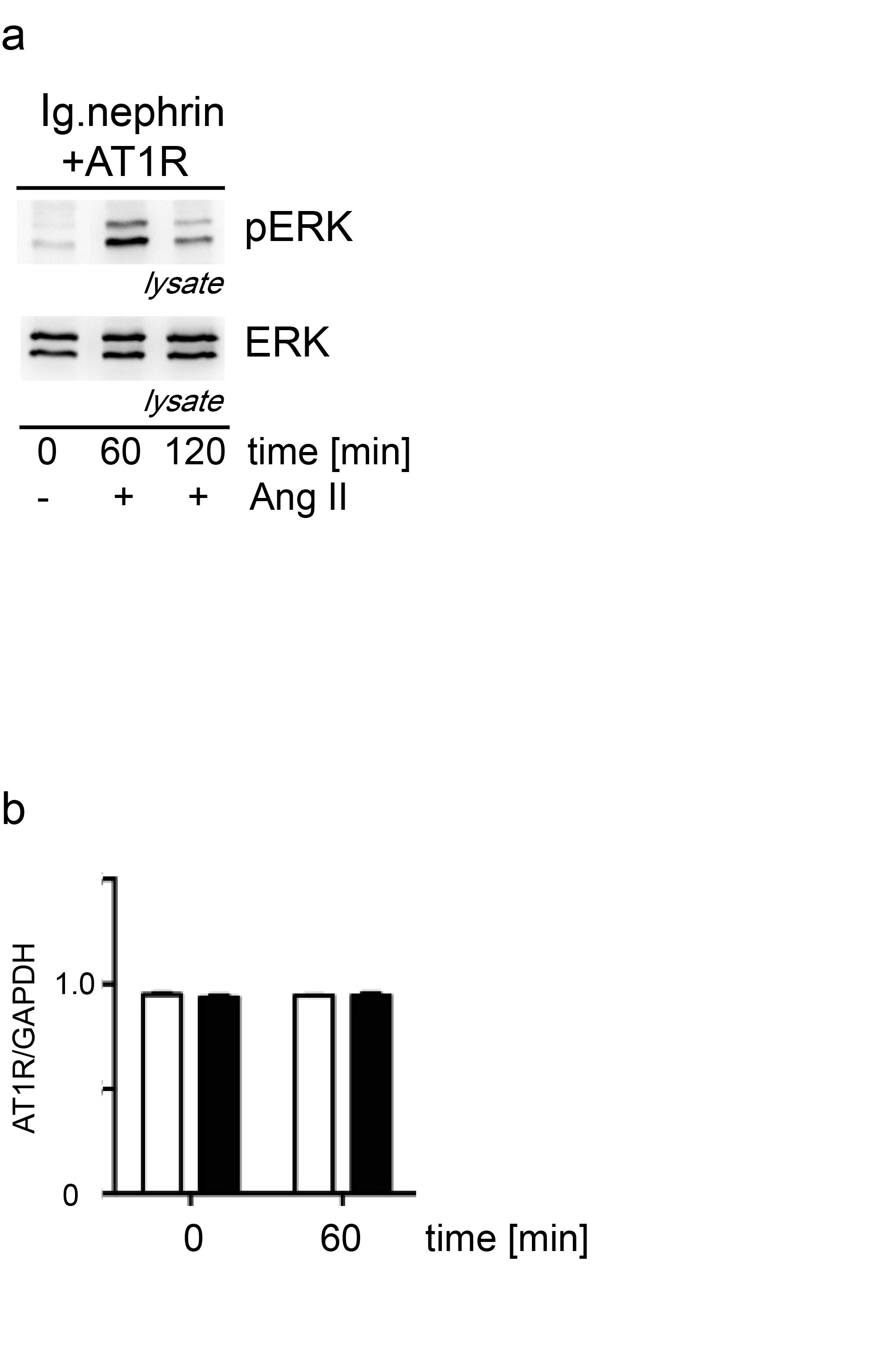
**
